# The impact of oral and systemic medications on the eye

**Published:** 2023-05-22

**Authors:** Shalinder Sabherwal, Abeer H A Mohamed-Ahmed

**Affiliations:** Director: Public Health and Projects, Dr Shroff's Charity Eye Hospital Network, New Delhi, India and Research Fellow: London.; Research Fellow in Pharmacology and Clinical Trials Management: London School of Hygiene & Tropical Medicine, UK.

**Knowing which systemic and oral medications can affect eye health, and how their adverse effects present, can help you to identify and manage patients correctly.**

**Figure 1:**
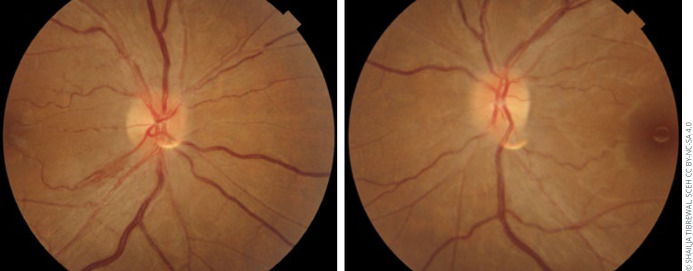
Bilateral toxic optic neuropathy with disc oedema in a 32-year-old female patient with a history of ethambutol intake and on tacrolimus after a renal transplant

A variety of oral and systemic medicines can have a harmful effect on the eyes.^1^ Some of the adverse effects may be dose-related, while others may not be.

Patients may not be aware of the relationship between the medication and their eye condition, and may not think to mention this to you, unless you specifically ask what medicines they are currently taking. They may not remember the name of their condition or the names of the medicine.

It is therefore helpful to be familiar with the different adverse effects of oral or systemic medications, so you can better identify and manage the eye condition the patient presents with. In addition to reporting the adverse reaction via the usual channels, it is advisable to contact the clinician who prescribed the medication so they can consider alternatives.

## Reporting of adverse drug reactions

There are national adverse drug reporting centres in 153 countries worldwide. Reporting of adverse reactions is mostly voluntary, and is done by health care professionals. At the global level, the World Health Organization Programme for International Drug Monitoring collates the reports from the national centres to ensure timely identification of suspected safety problems. To find out more, including how to set up an adverse drug reporting centre in your country, visit https://bit.ly/DrugWHO.

In addition to eye care professionals, physicians prescribing these drugs must also be made aware of any potential adverse reactions. That would enable them to warn the patients to report early symptoms and to undergo regular eye check-ups wherever indicated. A list of medications which can cause ocular toxicity is given in Table 1. Remember:

- Patients are unlikely to tell you what other medication they are taking, unless you ask.
- Some patients may not remember the name or dosage details, so you may need to check their records, if available.

**Table 1:** Drugs that can cause ocular toxicity.^1^

**Oral drugs**	**Use**	**Possible ocular side effects**
Topiramate	Treatment of epilepsy	Secondary angle-closure glaucoma, visual field defects, oculogyric crisis, uveitis
Gabapentin	Treatment of epilepsy	Nystagmus, diplopia and visual field defects
Vigabatrin	Treatment of epilepsy	Visual field constriction, optic nerve atrophy
Bisphosphonates (e.g., alendronate sodium,, risedronate, zoledronic acid	Treatment and prevetion of osteoporosis	Inflammation in the eye leads to conjunctivitis, episcleritis, scleritis, keratitis or uveitis, or corneal and scleral melting
Chloroquine-based drugs (chloroquine, hydroxychloroquine	Treatment of malaria	Maculopathy, peripheral retinopathy
Corticosteroids (e.g., prednisolone, dexamethasone)	Anti-inflammatory	Corticosteroid-induced raised intraocular pressure can lead to glaucoma, acceleration of cataract progression, and subcapsular cataracts.
Ethambutol	Treatment of tuberculosis	Optic neuropathy characterised by bilateral central visual loss, decreased colour vision, central visual field defects, and (eventually) optic atrophy
Fingolimod	Treatment of multiple sclerosis	Macular oedema, blurred vision, distortion, and impaired reading vision
Isotretinoin and vitamin A	Acne and vitamin A deficiency treatment, respectively	Blepharoconjunctivitis, chalazia, corneal opacities, dry eyes, retinopathy
Mitogen-activated protein kinase kinase enzyme (MEK) inhibitors, e.g., crizotinib	Treatment of advanced non-small cell lung cancer	Decreased visual acuity, visual field defects, dry eye symptoms, eyelid abnormalities, retinal vein occlusion, and retinopathy
Pentosan polysulfate	Relief of bladder pain and discomfort related to interstitial cystitis	Maculopathy, retinal pigment epithelial lesions
Phenothiazines	Treatment of schizophrenia and other psychotic disorders	Abnormal pigmentation of the eyelids, conjunctiva and cornea. Corneal epithelial changes (high dose). Corneal oedema (rare).
Phosphodiesterase type 5 inhibitors, e.g., sildenafil, tadalafil	Treatment of erectile dysfunction	Persistent blurred vision, non-arteritic ischaemic optic neuropathy, cilioretinal artery occlusion, or central serous chorioretinopathy.
Tamoxifen	Treatment of breast cancer	Intraretinal crystalline deposits, macular oedema, and punctate retinal pigmentary changes.
Tetracyclines, e.g., doxycycline, tetracycline	Antibiotics	Nausea, vomiting, and morning headaches may be symptoms of idiopathic intracranial hypertension which can lead to permanent loss of vision
Thiazolidinediones, e.g., glitazones, pioglitazone, rosiglitazone	Management of type 2 diabetes mellitus	Macular oedema

Some adverse reactions can affect vision and are potentially sight-threatening, while others may not cause loss of vision but can lead to hazy vision or discomfort.

## Sight-threatening adverse reactions

### Raised intraocular pressure

Patients may present with raised pressures in the eye caused by the intake of the following drugs.

- **Corticosteroids** (such as prednisolone or dexamethasone)^2^ are used as long-term medications for some joint disorders, skin diseases, auto-immune disorders and in transplant patients. They are administered by various routes: topically, orally, intravenous, nasally or as injections in joints and can raise intraocular pressure, resulting in secondary glaucoma.
- **Antihistamines, beta blockers, antidepressants, antipsychotics and some diuretics** can cause angle-closure glaucoma in pre-disposed patients who have shallow anterior chambers. These are drugs with anti-cholinergic effects and are used to treat conditions like urinary incontinence, chronic obstructive pulmonary disorder, allergies, or mental health conditions. This group of medications generally causes pupillary dilation leading to angle closure. Some sulfa-based drugs are also known to cause a similar reaction.^3^
- **Topiramate**, which is used to treat epilepsy, can cause uveal effusion with very high intraocular pressure. The symptoms include blurred vision, difficulty seeing, and eye pain, and usually happen in the first month of taking the medication.

Patients on these drugs must be monitored for intraocular pressure (IOP) and AC depth. Some patients are ‘high responders’ and can have significantly increased IOP. In case, corticosteroids cannot be tapered or replaced, IOP must be controlled medically.

### Cataract

- **Corticosteroids.** Long-term use of corticosteroids can also lead to posterior sub-capsular cataract. This form of cataract leads to vision-related issues early in its course and may warrant early surgery.
- Some other less-used drugs known to cause cataracts include **phenothiazines**, used for behavioural disorders and **busulfan**, an antineoplastic drug.^3^

### Toxic optic neuropathy

Patients with toxic optic neuropathy may present with bilateral, painless loss of vision (Figure 1). This has been reported with various drugs^4^:

- **Ethambutol** and **isoniazid**, which are commonly prescribed for tuberculosis in countries where tuberculosis is endemic; the risk of toxic optic neuropathy is greater in patients who also have renal disease
- **Ciprofloxacin** and **chloramphenicol**, both antimicrobial medications
- **Antimetabolite medicines** used in the treatment of malignancies
- **Amiodarone** used for arrhythmias
- **Amoebicidal medications**.

Patients on these drugs must be screened for visual acuity, colour vision, and central vision testing. The majority of these defects can be reversed with timely discontinuation and thus, timely monitoring is essential.

### Retinal haemorrhages and internal ocular bleeding

Bleeding in retinal tissues can lead to sight loss. This may be caused by the following medication:

- **Anticoagulants**^5^ used for the prevention of heart disease and stroke
- **Antineoplastic drugs** used for malignancies.

This can be monitored using blood tests and medication may need to be discontinued in some cases, especially where a minor bleed has already occurred. These drugs can also lead to bleeding during eye surgery and may have to be discontinued prior to some eye surgeries. It is therefore vital that eye surgeons know about patients’ usage of such drugs.

### Retinal toxicity

Some drugs can cause damage to one of the layers of the retina (retinal pigment epithelial loss). Unfortunately, some of these patients may already have central visual loss when they present. Retinal toxicity is irreversible, thus early recognition by regular screening, and early discontinuation, is imperative.

- **Chloroquine** and **hydroxychloroquine**^6^ are antimalarial drugs. They are more likely to cause retinal toxicity when used for longer periods of time, either to treat other inflammatory conditions of the joints or – more recently – as prophylaxes for COVID-19.
- **Thioridazine** and **chlorpromazine** are phenothiazines used for the treatment of anxiety, depression, and other behavioural disorders.

Check patients’ vision using manual or automated visual field testing or spectral-domain optical coherence tomography (if available). Multifocal electroretinogram (mfERG), if available, can be used for objective corroboration with visual fields.

### Other potentially sight-threatening adverse reactions to medication

**Figure 2:**
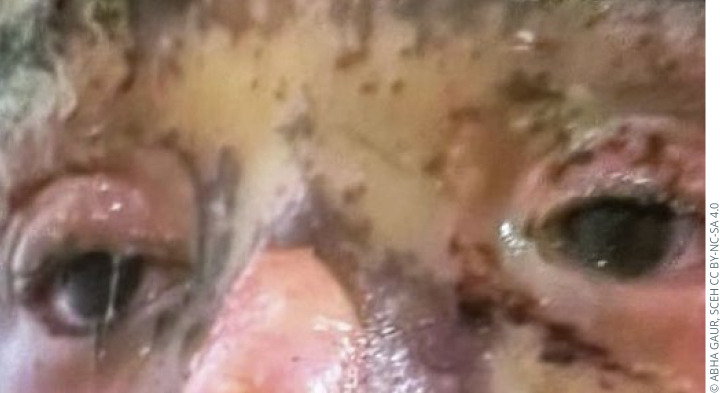
A 12-year-old child with acute Stevens-Johnson Syndrome. india

**Figure 3:**
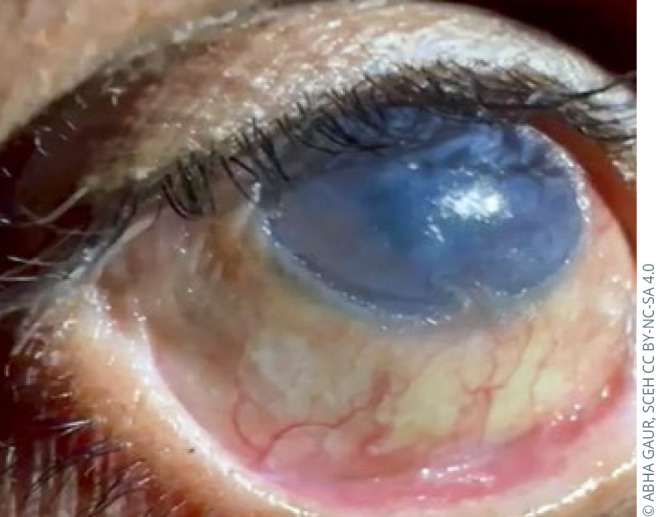
A 27-year-old man with sequelae of Stevens-Johnson Syndrome. INDIA

- **Central serous retinopathy.** Corticosteroids can cause central serous retinopathy (CSR) in some patients.
- **Intracranial hypertension.** Tetracycline, which is used long-term for conditions like rosacea can lead to intracranial hypertension or pseudotumor cerebri, which may lead to optic atrophy if left untreated.
- **Stevens-Johnson syndrome.** This is a relatively rare drug reaction, characterised by skin and mucosal involvement. It has an acute phase with severe pseudomembranous conjunctivitis (Figure 2) and a chronic phase with extreme dry eye and cicatricial features (Figure 3) and can be caused by common drugs such as painkillers or cold and flu medication. Over one hundred drugs have been associated with this syndrome.^7^ In the acute phase, treatment includes management of pain, topical and systemic anti-inflammatory medications, and antibiotics to control infection.

## Non-sight-threatening adverse reactions

These side effects may cause discomfort but may not be directly sight threatening.

### Corneal vortex keratopathy

This is a whorl-like pattern on the cornea and is generally not visually significant. These are mostly caused by amiodarone, a drug used to treat cardiac arrhythmia. Some of the other drugs which can cause this are chloroquine, hydroxychloroquine, indomethacin, and tamoxifen.^6^ The dosages need to be reduced only if the corneal condition causes extreme discomfort or blurring of vision.

### Floppy iris syndrome

Another specific drug-induced condition is one in which there is an effect on the constrictor muscles of the iris, leading to poor dilation and floppy iris during cataract surgery. This is generally caused by alpha-1 blockers like tamsulosin (used for prostatic hypertrophy).^8^ These technical issues during cataract surgery can be prevented if adequate precautions are taken, so patient's usage of these drugs must be known to the surgeon. It is sometimes recommended that tamsulosin be stopped two weeks before surgery, but it may be more important that the surgeon is made aware that the patient is taking one of these drugs.

### Dry eye

A diverse group of orally administered medications have been linked with dry eye. These include antihypertensive drugs such as atenolol and acebutolol, antihistamines such as cetirizine, antivirals such as aciclovir, analgesics (e.g., ibuprofen) and some antidepressants, antipsychotic, and anti-arrhythmic medications (see Table 1).^3^ Other conditions, like epiphora, blepharitis, and conjunctivitis can also be side effects of some anti-malignancy drugs that are administered systematically.^9^
